# Lipid-engineered nanotherapeutics for cancer management

**DOI:** 10.3389/fphar.2023.1125093

**Published:** 2023-03-23

**Authors:** Alicia Fernandez-Fernandez, Romila Manchanda, Manisha Kumari

**Affiliations:** ^1^ College of Healthcare Sciences, Nova Southeastern University, Fort Lauderdale, FL, United States; ^2^ Institute for Quantitative Health Science and Engineering, Michigan State University, East Lansing, MI, United States

**Keywords:** nanotechnology/nanomaterials, cancer, lipid, delivery systems, nanotherapeutics

## Abstract

Cancer causes significant mortality and morbidity worldwide, but existing pharmacological treatments are greatly limited by the inherent heterogeneity of cancer as a disease, as well as the unsatisfactory efficacy and specificity of therapeutic drugs. Biopharmaceutical barriers such as low permeability and poor water solubility, along with the absence of active targeting capabilities, often result in suboptimal clinical results. The difficulty of successfully reaching and destroying tumor cells is also often compounded with undesirable impacts on healthy tissue, including off-target effects and high toxicity, which further impair the ability to effectively manage the disease and optimize patient outcomes. However, in the last few decades, the development of nanotherapeutics has allowed for the use of rational design in order to maximize therapeutic success. Advances in the fabrication of nano-sized delivery systems, coupled with a variety of surface engineering strategies to promote customization, have resulted in promising approaches for targeted, site-specific drug delivery with fewer unwanted effects and better therapeutic efficacy. These nano systems have been able to overcome some of the challenges of conventional drug delivery related to pharmacokinetics, biodistribution, and target specificity. In particular, lipid-based nanosystems have been extensively explored due to their high biocompatibility, versatility, and adaptability. Lipid-based approaches to cancer treatment are varied and diverse, including liposomal therapeutics, lipidic nanoemulsions, solid lipid nanoparticles, nanostructured lipidic carriers, lipid-polymer nanohybrids, and supramolecular nanolipidic structures. This review aims to provide an overview of the use of diverse formulations of lipid-engineered nanotherapeutics for cancer and current challenges in the field, as researchers attempt to successfully translate these approaches from bench to clinic.

## 1 Introduction: Nano approaches in cancer management

**FIGURE 1:**
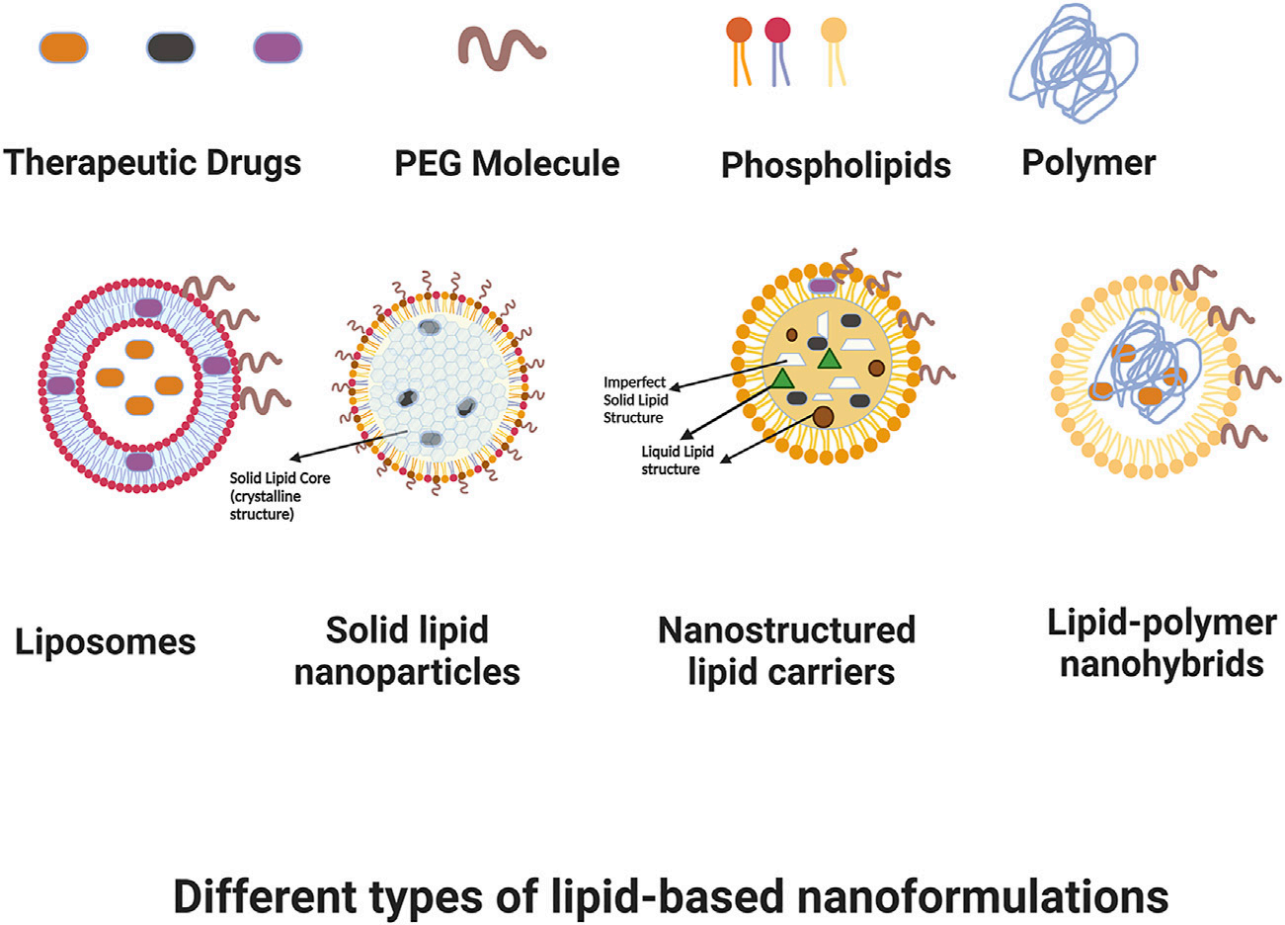
Some types of lipid-based nanoformulations. Created with BioRender.com.

Cancer, as a very heterogeneous disease, has proven to be difficult to effectively understand and therefore manage and treat. Although researchers have worked for many decades on developing therapeutic strategies for cancer, and survival has certainly improved in recent years, there continue to be many challenges in scaling treatments that are selective and minimize off-site negative effects, while efficaciously targeting cancer cells with chemotherapy, radiotherapy, hyperthermia, and other therapeutic modalities. Overcoming difficulties with toxicity, poor pharmacokinetics or bioavailability, low effective dosage, resistance profiles, and the intricacies of the cancer microenvironment all continue to be a focus of research and development efforts. Nanoformulations have shown a distinct advantage in these applications because of their customizability. Nano structures can be rationally-designed and surface-engineered to achieve longer plasma half-lives and improved biodistribution compared to traditional drug approaches by modifying their interactions with plasma components, and their nano size allows them to preferentially target tumors through a leaky vasculature environment, with this passive accumulation in the tumor interstitial space potentially reducing off-target toxicity. Additionally, these structures can be further targeted in an active manner; for example, being customized to interact in a specific fashion with receptors that are overexpressed in cancer cells in order to achieve enhanced target uptake; or targeting to tumor vasculature in order to decrease neoangiogenesis and/or vascularization that is critical to tumor survival. Nanoformulations can also have responsive therapeutic delivery or deployment, with actions that are triggered based on certain conditions such as temperature and heat once they reach the desired target. In drug applications, release can be controlled based on the customization of nanoformulation components. Finally, nanoformulations are not limited to a single specific mechanism of action, but rather they can be designed as multifunctional theranostic platforms that have potential for diagnosis, monitoring, and combined therapeutic approaches; for instance nanoformulations that allow for imaging, hyperthermia, and chemotherapy in a single design, among other examples. Among the different nanoformulations, lipid-based structures are one of the most researched and utilized due to their reduced toxicity for *in-vivo* applications. In this review, we aim to present an overview of lipid-based nanoformulations and how they have been developed and utilized for use in cancer management. Figure 1 shows some of the main types of lipid-based nanoformulations that we will be discussing.

## 2 Lipid-based nanoformulations for cancer treatment: Design, preparation, and applications

### 2.1 Liposomes

Bangham et al. were the first to report that lipids create bilayer structures in an aqueous medium, (Bangham and Horne, 1964), and these structures were given the name “liposomes” in 1968 by Sessa and Weissmann (1968). Liposomes consist of an aqueous core surrounded by phospholipid bilayers, with the hydrocarbon chains in the interior and the polar heads oriented facing the aqueous medium. Liposomes, based on this structure, can be used to entrap different therapeutic drugs and to help deliver them to target sites. Different types of phospholipids can be used in liposomal formulations, such as natural phosphatidylcholine (from egg or soy), synthetic phosphatidylcholine (PC), phosphatidylenolamine (PE), and phosphatidylserine (PS). The most commonly used form of PC in liposomal formulations is (2- distearoyl-sn-glycerophosphocholine (DSPC).

Liposomes can be classified based on their preparation techniques, size, and number of bilayers. Generally liposomes are categorized as multilamellar vesicles (MLVs), which range in diameter between 0.05 and 10 µm and have many concentric lipid bilayers; and unilamellar vesicles (ULVs), which have only one bilayer and can be further subdivided into large unilamellar vesicles (LUVs, with diameter greater than 100 nm) and small unilamellar vesicles (SUVs, with diameters up to 100 nm). (Masserini, 2013). Liposome preparation methods can be mechanical (film method, ultrasonication method), based on replacement of organic solvent (reverse-phase evaporation method, ether vaporization method), or based on size transformation or fusion of preformed vesicles (freeze-thaw extrusion method, dehydration-rehydration method). (Ateeq et al., 2018). Several methods are typically combined to optimize the desired formulation. The most commonly used methods in initial preparation are the film and solvent evaporation methods. Briefly, lipids having 10–20 mg/mL concentration are dissolved in a chloroform/methanol mixture, and then solvents are removed using a rotary evaporator to achieve a very thin lipid film which is then hydrated by aqueous solution. The resulting MLVs are usually in the bigger size range, so sonication and extrusion methods are then utilized to further break down these MLV into smaller sized liposomes in the desired range. A water bath sonicator set at a temperature above the phase transition temperature (Tc) of lipids is used to perform sonication, which creates sonic waves that disrupt the outer layer of MLVs and result in smaller size SUVs. The exact size will vary depending on sonication time, energy, lipid composition, and concentration. Extrusion can also be used to reduce the size of MLVs. This method consists of maintaining high pressure and temperature above the lipid Tc, while the MLV solution is passed through polycarbonate membrane filters having a specific pore size, so that the final size of the extruded liposomal formulation depends on the pore size of the membrane used. The phase transition temperature (Tc) is a critical criterion to consider when preparing liposomes, not only because it needs to be considered for mechanistic reasons during sonication and extrusion processes, but also because it greatly impacts liposome stability. The Tc is the temperature at which the physical state of a lipid changes from an ordered gel phase to a disordered liquid crystalline phase. Phase conversion depends on hydrophobic chain length, saturation degree, charge, and other physicochemical factors. Phospholipids having higher Tc create stable bilayers, and have reduced risk of premature leakage of encapsulated agents. However, phospholipids with too high Tc may lead to degradation of entrapped drugs during the formulation process, so selecting lipids with proper Tc is important to achieve stability while avoiding undesirable changes in the therapeutic load.

The *in vitro* and *in vivo* stability of liposomes depends largely on their size, charge, lipid configuration, and additional components. Cholesterol is commonly added to liposomal formulations to stabilize the bilayer. The hydroxyl groups of cholesterol face towards the aqueous phase of the phospholipid molecules, and the hydrophobic ring inserts into the initial few carbons of acyl chains in the hydrocarbon center of the bilayer membrane. The combination of cholesterol with phospholipids enhances the stability of liposomes and increases the viscosity of liposomal membranes. Additionally, cholesterol reduces the permeability of hydrophilic agents across the liposomal interface. Being a hydrophobic molecule, cholesterol sits in the internal side of the lipid bilayer and helps seal the gaps produced by imperfect packaging of phospholipid molecules, thus minimizing movement and transport through the layers. In the absence of cholesterol, liposomes destabilize easily in biological fluids due to interactions with blood elements and plasma proteins, such as albumin or transferrin. (Singh et al., 2018). Other elements can be added to liposomes to improve their *in vivo* stability. Polyethylene glycol (PEG), a small polymer, stabilizes a liposome when added to its formulation because it creates steric hindrance against the interaction of the liposomal lipid layers with plasma proteins. A variety of PEG-based liposomal formulations have been used in various applications, (Yatuv et al., 2010; Mohamed et al., 2019), with the most common being combinations of PEG with functional phospholipids such as 1,2-distearoylsn-glycero-3-phosphoethanolamine (DSPE). Chitosan, a naturally-derived polymer, has also been utilized to improve the stability of liposomes, and can be incorporated into liposomes using ionic interactions between the negatively charged liposomal surface and the positively charged hydrophilic chitosan. The charge, shape, size, and pharmacokinetics of the final liposomal formulation are dependent on the components chosen, particularly the molar percentage and packaging orientation of the phospholipids, as well as the additional constituents that may determine surface charge and interactions with biological systems.

Liposomal drug delivery offers numerous benefits compared with conventional therapeutics, such as reduced systemic effects of the encapsulated drug, more controlled pharmacokinetics, predictable drug release profiles, and passive and active tumor targeting. Drug encapsulation into liposomes for delivery purposes can be achieved using two methods: passive and active. Passive loading involves the entrapment of the therapeutic load during liposome formulation, and the process largely depends on the chemical characteristics of the drug, particularly its hydrophobicity or hydrophilicity. For instance, a hydrophilic drug can be mixed with the hydrating buffer in the hydration step during formulation, whereas a hydrophobic drug can be mixed with phospholipids during the formation of the thin dry film of lipids, so that it is entrapped into the lipid layer. Passive methods usually lead to low encapsulation efficiency for hydrophilic drugs, given their limited interaction with lipid layers. Encapsulation efficiency of these drugs can be improved by conjugating a hydrophobic chain to the drug molecule, thus achieving enhanced partition into the lipid bilayer. (Bozzuto and Molinari, 2015). However, bioactivity may be affected by conjugation, so any modification must be carefully considered and reevaluated for therapeutic efficacy. Lipid selection is also a critical factor in order to improve hydrophilic drug entrapment. Negatively-charged agents such as DNA or siRNA will show better entrapment in the presence of cationic lipids due to enhanced drug/lipid interaction.

Active loading, also known as remote loading, involves drug loading after the liposome has been formed. A potential gradient (pH or ionic gradient) is created between bilayers of liposomes using different buffers with specific pH and ionic concentrations, and the outside pH/ionic concentration is exchanged with another buffer or different concentration using dialysis, or size exclusion chromatography. Once the gradient is created among liposome membranes, the drug is incorporated by mixing liposomes at a temperature higher than the Tc of the lipids. Liposome and drug interaction results in charged drug-loaded carriers, where drugs stay within the core of the liposomes and will not leach out. Doxil™, liposomal doxorubicin, is an example of active loading using a pH gradient method, and PEGylated liposomal doxorubicin was the first United States FDA-approved liposomal formulation for cancer applications. (Barenholz, 2012).

A suitable amount of drug encapsulation is important to have a proper therapeutic effect, and it can be achieved as described above through active and passive loading methods, while considering the hydrophilicity or hydrophobicity of the drug. However, encapsulation efficiency of drugs depends not only on the nature of the drugs, but also on the lamellarity of the liposomes. MLVs having manifold phospholipid bilayers show comparable or higher encapsulation efficiency than LUVs and SUVs. Pignatello et al. showed that 2 mm or large-sized MLVs had encapsulation efficiency of luteinizing hormone of around 78.8%–81.4%, whereas LUVs in the 241.0–269.5 nm size range demonstrated 66.7%–77.9% entrapment efficiency of the same hormone. (Pignatello et al., 2016).

Even when a desirable payload amount has been achieved and the formulation is shelf-stable, potential biological interactions must be taken into account when successfully designing liposomal carriers for *in vivo* applications. Liposomes can be administered using various routes, such as intravenous, oral, and topical. Oral delivery of liposomes is seriously hindered by poor stability in the gastrointestinal tract and low penetration across the intestinal epithelium. (Huwyler et al., 2008; He et al., 2019) Most of the liposomal formulations that have achieved clinical translation are administered parenterally, particularly intravenously. The reticuloendothelial system (RES) is comprised of different cells and tissues which assist in monitoring and eliminating foreign substances by filtration and interception in plasma, spleen, liver, lungs, and lymph nodes, among others. Carrier size is an important factor in determining interactions of intravenously administered agents with the RES. Researchers have reported that large sized liposomes (around 330 nm) are more easily filtered by the spleen than smaller-sized particles in the range up to 200 nm. (Litzinger et al., 1994; Awasthi et al., 2003). Moreover, Awasthi et al. showed a direct relationship (*R* ^2^ = 0.98) between liposomal size and spleen uptake in a rabbit model. It has also been observed that liposomes that are 200 nm or higher, even with PEG coating, show faster rates of intravascular clearance through splenic filtration when compared to carriers with sizes under 200 nm. Wacker et al. have suggested that colloidal size for intravenously injected formulations should be between 100 and 300 nm to have optimal circulation time and enhanced tumor accumulation, although of course many other physicochemical factors such as surface charge need to also be taken into consideration. (Wacker, 2013). Additionally, clearance of liposomes by the RES after opsonization with serum proteins is a known issue, although it can be ameliorated by PEG decoration. Another advantage of formulations containing PEG is that they minimize the tendency of liposomes to aggregate, (Bozzuto and Molinari, 2015), which would otherwise result in premature release of the payload. Thus, PEGylation contributes to longer circulation times as well as a more controlled release profile.

A stable, long-circulating carrier still has to overcome the challenge of being delivered to a target site in sufficient amounts. Liposomes can utilize the EPR effect for passive accumulation in tumors, but can also be designed to actively target tumors in a tailored manner by interacting with receptors that may be overexpressed by tumor cells, or by modulating gene expression or receptor function in the tumor endothelium. For instance, liposomes have been widely used for targeted applications in gastric cancer, associated with molecules such as Arg-Gly-Asp peptide to target integrin receptors, (Ding et al., 2015), SATB1 siRNA/CD44 antibodies, (Yang et al., 2018), or by forming DNA complexes. (Wonder et al., 2018). PEGylated liposomes, modified with an Arg-Gly-Asp peptide, have been shown to increase drug accumulation in tumor-bearing mice transplanted with gastric cancer SGC7901 cells which overexpress integrin α5β1. (Ding et al., 2015). Liposomes have also been utilized to target tumor endothelium. After intravenous administration, liposomes encounter the glycocalyx at the endothelial surface of the cells prior to getting into the interstitial space. The negatively charged glycocalyx of angiogenic endothelial cells can associate with positively charged liposomes. For instance, a positively-charged liposomal formulation loaded with camptothecin (Endo TAG-2) showed decreased microvessel density of up to 50% in a mouse model of LLC-1 lung carcinoma. (Eichhorn et al., 2007). Liposomes can also be used for specific targeting of endothelial-related targets. For instance, vascular endothelial growth factor (VEGF) and kinesin spindle protein (KSP) siRNA-loaded liposomes were administered to immunodeficient mice implanted with Hep3B human hepatocellular carcinoma cells. Administration of this therapeutic formulation resulted in dose-dependent reduced gene expression of VEGF and KSP, with up to 50% reduction in 24 h, and an associated decrease in tumor perfusion and hemorrhage, as well as incomplete mitosis due to KSP reduced expression. In a subsequent phase I clinical trial in patients with liver tumors, the same formulation resulted in decreased expression of VEGF and a related decrease in tumor blood flow in nearly half of the patients. (Tabernero et al., 2013). Another example of a liposomal formulation that targets endothelial function is Arg-Gly-Asp(RGD)-conjugated PEGylated doxorubicin (DOX)-loaded liposomes. RGD is an oligopeptide that can be used to target αvß3 integrin, a highly expressed tumor cell protein that acts as an endothelial cell receptor and is essential for calcium-dependent pathways involved in endothelial cell migration. RGD-conjugated PEGylated DOX-loaded liposomes demonstrated a significant therapeutic effect with up to 50% reductions in tumor volume when administered in a mice model of DOX-insensitive C26 colon carcinoma. (Schiffelers et al., 2003).

An additional aspect of targeting and maximizing effective delivery to desired sites is the use of stimuli-responsive liposomes, which can be used to selectively release their payload in the presence of specific triggers. A classic example is the use of liposomal formulations with pH-sensitive lipids such as PE or PC, which release their load once they reach the acidic microenvironment of tumors. Wang et al. (2014) used cytarabine-loaded liposomes with an octylamine-implanted polyaspartic acid moiety, and showed that this formulation had an enhanced antitumor effect and toxicity in human HepG2 hepatoma cells, while reducing toxic effects in normal human liver L02 cells. In another study by Tripathy et al. (2015), nanocomplexes were created that combined pH-sensitive zinc-oxide nanoparticles and a daunorubicin-loaded liposomal formulation. Zinc-oxide nanoparticles dissociate under acidic conditions and trigger the release of liposomal encapsulated drug. These nanocomplexes were more cytotoxic to alveolar adenocarcinoma A549 cells when compared to a daunorubicin-loaded liposomal formulation without a triggered release mechanism. Another example of tailored payload release is the use of temperature-sensitive liposomal formulations that utilize regional heating of cancer cells for enhanced drug delivery (Needham et al., 2013; Borys and Dewhirst, 2021). Heating modalities that are used to trigger release from thermosensitive liposomes can vary and may include radio frequency heating, focused microwaves, infrared laser, ultrasound, and high-intensity focused ultrasound (HIFU). One key component in temperature-sensitive liposomal formulations is 1,2-dipalmitoyl phosphatidylcholine (DPPC), which can change from gel to liquid phase at 41°C and release the incorporated payload (Jeong et al., 2009). Researchers have also introduced other moieties in temperature-sensitive liposomal formulations, such as 1,2distearoyl-sn-glycerol-3-phosphocholine (DSPC) or 1-steroyl-2-hydroxy-snglycero-3-phosphocholine (MSPC), to enhance permeability and release under mild hyperthermic conditions, and there have been recent advances in designing target-specific vehicles, such as the combination of drug-loaded thermosensitive liposomes with gene-engineered exosomes to enhance tumor targeting (Cheng et al., 2021; Chaudhry et al., 2022). More recently, Liang et al. (2023) developed a micelle-liposome hybrid nanoparticle platform to treat multidrug resistant cancer using DOX and si/RNA co-loading, which was pH-responsive and enhanced cytotoxicity in a breast cancer line. The formulation also decreased tumor growth in a mouse model of multi-drug resistant breast cancer, while reducing cardiotoxic effects.

Some more detailed examples of liposomal formulations are shown in Table 1.

**TABLE 1:** Examples of liposomal formulations.

Liposomal composition	Targeting (agents)	Drug	Size (nm)	Remarks	References
1,2-Distearoyl-sn-glycero-3-phosphocholine (DSPC), Cholesterol, FA-PEG2000-DSPE	Folate (FA)	Bleomycin	100–150	Enhanced targeted FL-BLM uptake by Hela cells as compared to non-targeted liposomes	Chiani et al. (2018)
Cholesterol,1,2-Dipalmitoyl-sn-glycero-3-phosphocholine (DPPC),1,2-distearoyl-sn-glycero-3-phosphoethanolaminse-N-[amino (polyethylene glycol)-2000] (ammonium salt) (DSPE-PEG2000 Amine)	EGFR	DOX hydrochloride	150–200	Targeted thermosensitive liposomes selectively targeted EGFR-positive tumors *in vitro* and in xenograft mice	Haeri et al. (2016)
Soya phosphatidyl choline, Distearoylphosphatidylethanolamine (DSPE)	Folate (FA)	5-Fluorouracil	100–150	Targeted liposomal 5FU showed enhanced *in vivo* antitumor effect in comparison to free drug	Moghimipour et al. (2018)
Egg phosphatidylcholine (ePC), cholesterol, cholesteryl hemisuccinate (CHEMS), 1,2-dioleoyl-sn-glycero-3-phosphoethanolamine (DOPE), 1,2-distearoyl-sn-glycero-3-phosphoethanolamine-N-[methoxy (polyethylene glycol)-2000] (PEG), (lissamine rhodamine) DPPE (rhodamine)	Transferrin and Folate	DOX hydrochloride	150–200	Dual-targeted liposomes showed tumor inhibition in HeLa xenograft model in nude mice, and these liposomes were as effective as folic acid-targeted liposomes in decreasing tumor burden	Sriraman et al. (2016)
1-palmitoyl-2-oleoyl-sn-glycero-3-phosphocholine (POPC), 1,2-dioleoyl-sn-glycero-3-phosphoethanolamine (DOPE), 1,2-distearoyl-sn-glycero-3-phosphoethanolamine-N-[methoxy (polyethylene glycol)-2000] (DSPE-PEG2000), and cholesterol	RNA aptamer (prostate specific membrane antigen)	DOX hydrochloride	100–150	Dox-encapsulating aptamer bearing liposomes were more toxic to the targeted prostate cancer cells than to non-targeted cancer cells, and showed improved antitumor efficacy *in vivo*	Baek et al. (2014)
1,2-dipalmitoyl-sn-glycero-3-phosphocholine (DPPC), 1,2-Distearoyl-sn-glycero-3-phosphoethanolamine-N-methoxy (polyethyleneglycol) (DSPE-PEG2000), Cholesterol, dimethyldioctadecyl-ammonium bromide (DDAB)		Docetaxel + siRNA	130–170	Co-delivery of Docetaxel/BCL-2 siRNA pegylated liposomes efficiently inhibited tumor regression in A549 cell bearing xenograft tumor mice model	Qu et al. (2014)
1,2-dipalmitoyl-sn-glycero-3-phosphocholine (DPPC); 1,2-distearoyl-sn-glycero-3-phosphoethanolamine-N-(amino (polyethylene glycol)-2000) (DSPE-mPEG2000), and myristoyl hemolytic lecithin (MSPC)		Purine derivative F7+Topotecan	100–150	F7 and Topotecan co-loaded thermosensitive liposomes showed strong tumor inhibition when combined with hyperthermia in both *in vitro* (Human lung large cell carcinoma) (NCI-H460 cells) and *in vivo* xenograft tumor model of nude MCF-7 bearing mice	Du et al. (2020)
1,2-dioleoyl-3-trimethylammonium-propane chloride (DOTAP), 1,2-distearoyl-sn-glycero-3-phosphoethanolamine-N-[methoxy-(polyethylene glycol)-2000] (PEG-DSPE), cholesterol and egg phosphatidylcholine (ePC)	Oleic acid modified R8 Peptide	Doxorubicin hydrochloride	90–100	Peptide-based doxorubicin- loaded liposomes showed better BBB penetration capability with enhanced cellular uptake, antitumor effect in *in vivo* glioma model	Yuan et al. (2019)
Soybean phosphatidylcholine, Cholesterol,1,2-distearoyl-sn-glycero-3-phosphoethanolamine-N-[maleimide (polyethyleneglycol)-2000](DSPE-PEG2000-Mal)	Protein TAT (Cys-AYGRKKRRQRRR)	Paclitaxel	100–120	PTX-loaded exogenous-GSH triggered TAT-presenting liposomes (PTX-C-TAT-LP) showed superior antitumor effect both *in vitro* and *in vivo*	Fu et al. (2015)
Egg yolk phosphatidyl choline (EYPC), l-dioleoyl phosphatidylethanolamine (DOPE)		Ovalbumin	100–150	pH Sensitive dextran modified liposomes, MGlu-Dex-, were taken up by dendritic cells and delivered their contents efficiently into the cytosol	Yuba et al. (2014)

### 2.2 Lipidic nanoemulsions

Nanoemulsions (NEs) are the colloidal dispersions of oil and water systems, which can be used as drug delivery vehicles, mainly for drugs having low water solubility. (Sánchez-López et al., 2019). These are composed of stable heterogeneous dispersions of nanometer sized droplets in another liquid, which provide high solubility to the drugs. NEs are reported to protect the drug from degradation and increase its plasma half-life. Emulsifying agents are used to stabilize the NEs. Emulsifying agents are amphiphilic compounds which reduce the interfacial tension between two immiscible phases, generally consisting of hydrophobic tails which place themselves in non-polar liquids and a polar head which tend to place itself in polar liquids. The droplet size of the NEs is usually in the range of 50–200 nm depending on the composition and method of production (Ganta et al., 2014) The emulsion can be oil-in- water or water-in- oil, basing on whether the dispersed phase is oil or water, respectively. NEs are usually prepared from excipients which are Generally Recognized as Safe (GRAS) and approved by Unites States Food and Drug Administration (FDA).

NEs can be easily produced in large quantities by mechanical extrusion and high shear stress, utilizing various techniques such as high-pressure homogenization, microfluidization, ultrasonication, and titrimetric methods (Chavda, 2019). The phase behavior of the NE is mainly governed by the type of the surfactant used. Flocculation, creaming, sedimentation, and coalescence are some of the characteristics which indicate the destabilization of the NEs. Long lived NEs are considered thermodynamically stable. Selection and order of the components, and method of shear are the important factors for getting stable NEs. The surfactant amount can be roughly estimated by assuming its equilibrium surface density on the droplet interfaces.

NEs have been explored for enhanced drug solubility and bioavailability and increased cellular uptake (Chavda, 2019). NEs are also reported to mitigate the toxicity associated with ethoxylated castor oil and the surfactants (Ganta et al., 2014). NEs have been used widely for drug delivery of anticancer therapeutics. The nanosized droplets can be easily targeted to tumor tissues by conjugating some targeting moieties on the surface of the NEs. Several studies indicated the use of colloidal carriers for delivery of various anticancer therapeutic agents (Ganta et al., 2010; Talekar et al., 2012; Kolluru et al., 2021; Mu et al., 2022). Sai et al. prepared lipidic nanoemulsions (LNEs) composed of triglyceride (medium chain length), lecithin, soybean oil and doxorubicin. The LNEs proved to be safe and were equally effective as that of commercial liposomal formulation Adriamycin (Jiang et al., 2013). Doris et al., developed a novel photosensitizer (PS) encapsulated in m-tetrahydroxyphenylchlorin (mTHPC) nanoemulsion. They compared the nanoemulsion formulation with a liposomal mTHPC formulation (Foslip) and found that nanoemulsions have superior biocompatibility compared to the liposomal formulation (Hinger et al., 2016).

Formulations employing NEs have also been utilized to obtain a synergistic effect between two therapeutic agents. For instance, Xi et al. developed a lipid NE for co-encapsulation of doxorubicin and bromotetrandrine to reverse multidrug resistance in breast cancer (Cao et al., 2015). Li et al. (2022) fabricated lipid NEs for co-delivery of paclitaxel (PTX) and docosahexaenoic acid (DHA). These NEs reduced tumor volume, prolonged survival, and reduced *in vivo* toxicity in an animal model.

In addition to their tumor therapeutic potential, NEs have also been explored as diagnostic agents. (Gianella et al., 2011). For instance, Fernandes et al. (2016) developed perfluorohexane nanoemulsions, which played a dual role as drug delivery vehicles and contrast agents for cancer photoacoustic imaging and ultrasound *in vivo*, offering deeper penetration depth and higher spatial resolution compared to conventional optical techniques. Wu et al. (2017) employed a similar strategy for developing magnetic NE hydrogels which induced magnetic hyperthermia-based tumor regression. More recently, Asmawi et al. (2023) prepared tunable-size docetaxel and curcumin-loaded nanoemulsions, which demonstrated good aerodynamic pulmonary delivery properties and reduced toxicity in human lung fibroblast (MRC-5) cells.

Several NE-based drug formulations are in the pharmaceutical market, and many others are in various stages of clinical development. (Kishore et al., 2022; Moghassemi et al., 2022).

### 2.3 Solid lipid nanoparticles

Solid lipid nanoparticles (SLNs), also known as lipospheres or solid lipid nanospheres, are particles in the range of 50–1000 nm that are prepared from lipids which stay in solid form at both room and body temperature. SLNs are formulated as a submicron sized lipid emulsion where the liquid lipid (oil) has been replaced by a solid lipid. They are composed of solid lipid, emulsifier, and water/solvent. Examples of lipids used in these formulations include triglycerides (tri-stearin), partial glycerides (which are esters of glycerol with fatty acids), fatty acids (stearic acid, palmitic acid), steroids (cholesterol), and waxes (cetyl palmitate) are used as matrix material for drug encapsulation. Stabilizers such as Pluronic F 68, and Pluronic F127 are utilized to stabilize the lipid dispersion.

Preparation of SLNs involves a dispersed system as precursor or template, or the use of specific instrumentation such as spray-drying, spray-congealing, and electrospray. Most precursors are emulsions which can be used for SLN preparation. Lipids that are solid at room temperature can be heated to above their melting temperature to obtain a liquid lipid that can be emulsified with an aqueous solution, and finally the drops can solidify in the form of SLNs. Methods utilized in the preparation of SLNs include hot/cold high-pressure homogenization, microemulsion, and precipitation of lipid particles by solvent evaporation. Homogenization involves extreme temperatures (either hot or cold) and high pressures of 100–200 bars, maintained while lipids are forced to pass through a narrow space of limited micron ranges. Shear stress under these specific temperature and pressure conditions gives rise to small range particles. The microemulsion method utilizes lipids with low melting points, emulsifier(s), co-emulsifier(s), and water. During the stirring process, the hot microemulsion is dispersed in cold water (2°C–3°C) at a microemulsion:water volume ratio of 1:25 to 1:50. The lipid phase precipitates out and gives rise to smaller particles, and an ultrafiltration method is used to remove the excess water and emulsifier. In the method of solvent emulsification-evaporation, hydrophobic components are dissolved in organic solvent and then further emulsification is performed in water using a homogenizer for uniform reduction in particle size. Then, organic solvents are evaporated using a stirrer or rotatory evaporator, with the SLNs as final product. This process has the advantage of preventing any thermal stress, so it is the preferred method when incorporating thermolabile drugs into SLNs. Other methods involved in the preparation for SLNs are solvent emulsification-diffusion method, ultrasonication, melting dispersion method (hot melt entrapped method), double emulsion approach, and spray drying technique (Thulasi Ram et al., 2012).

SLNs have important advantages as nanocarriers in cancer therapy, such as their low toxicity, high drug bioavailability, versatility of incorporation of hydrophilic and lipophilic drugs with high entrapment efficiencies, and potential for sustained drug release (Mishra et al., 2010). SLNs could be particularly suited for oral administration of drugs, which could be more convenient for patients and could enhance the ability to maintain sustained drug dosage exposure (Mei et al., 2013). Several groups have studied the effect of size, zeta potential, and material constituents on oral bioavailability of drugs (Gao et al., 2017; Tian et al., 2017). In one study done by Shi et al. (2017), cationic charged chitosan or HACC-SLNs loaded with docetaxel (DTX) demonstrated enhanced oral absorption by electrostatic attraction with cells. Oral SNL formulations loaded or coupled with anticancer agents have been studied in animal models. In one study, SLNs coupled with estrogenic derivative (ESC8), administered orally, demonstrated good bioavailability (47% of dose) and resulted in 74% reduction in breast tumor growth in mice with MDA-MB-231 breast cancer xenografts compared to controls. The formulation also enhanced the effect of the chemotherapy drug cisplatin when administered concurrently (Andey et al., 2015). In another study, Jain et al. (2013) prepared SLNs loaded with quercetin, which were showed increased anti-tumor activity against B16F10 melanoma cells in C57BL/6 mice as compared with a quercetin suspension.

Other administration routes such as intravenous are also feasible for SLN formulations, and their small size facilitates escape from RES components (Pardeshi et al., 2012; Correia et al., 2022). Zheng et al. (2019) prepared pH-sensitive DOX loaded SLNs (RGD-DOX-SLNs) for the treatment of breast cancer, and reported that this formulation showed a 5.5 fold higher area under the plasma concentration-time curve when compared to a DOX solution, after intravenous administration in a MCF-7/ADR breast cancer mouse model. SLNs have also shown promising results for brain tumor drug delivery, and may demonstrate an enhanced ability to cross the blood-brain barrier (Blasi et al., 2007; Gastaldi et al., 2014; Satapathy et al., 2021). Kharya et al. (2013) developed phenylalanine-coupled SLNs loaded with ionically-complexed DOX, which demonstrated enhanced uptake by glioma cells. Song et al. created borneol-modified chemically solid lipid nanoparticles (BO-SLN/CM) of about 87-nm diameter, which demonstrated enhanced blood–brain barrier permeability *ex vivo* and *in vivo* based on fluorescence imaging, when compared with SLNs alone (Song et al., 2018). Wang reported that 3′,5-dioctanoyl-5-fluoro-2-deoxyuridine (DO-FUdR) incorporated in SLNs improved *in vivo* brain-targeting efficiency by about 2-fold compared to free FUdR (Wang et al., 2002). While the mechanism by which SLNs cross the blood-brain barrier is still unidentified, it is believed that their small size allows them to cross tight junctions, perhaps by endocytosis (Pardeshi et al., 2012). In other tissues, the endocytosis process has been shown to be facilitated by the adsorption of blood plasma proteins onto the SLN surface (Nagayama et al., 2007).

Some disadvantages of SLNs include recrystallization processes, low drug encapsulation, and the probability of drug release during SLN storage. Additionally, some SLN formulations show high polydispersity, which may be a barrier for the large-scale synthesis of SLNs. (Sheoran et al., 2022).

### 2.4 Nanostructured lipidic carriers

Nanostructured lipidic carriers (NLCs) were designed to improve on SLNs by enhancing drug loading capacity and providing increased trapping stability of drugs within the matrix. In NLCs, drugs are incorporated in a combination of solid lipid and liquid lipid phases, with changing ratios. Also, the NLC matrix is made to be less crystalline (or not at all) during solidification, in contrast to the crystalline core of SLNs. The methods of preparation of NLCs are thus similar to the ones discussed earlier for SLNs, with the only difference being the constitution of the core/matrix. In SLNs, drugs are mainly liquefied in solid lipids, whereas NLCs are prepared by having the drug solubilized or melted in the liquid and solid lipid blend, and then dispersed in aqueous medium with emulsifier. In the process of homogenization and in storage, the SLN core forms an orderly-fashioned crystalline structure. This structure results in less space available for drugs, and in some cases may lead to drug leakage from the dispersion medium and drug load losses during preparation or while in storage. In contrast, the combination of liquid and solid lipids in NLCs results in an amorphous structure of the drug-loaded matrix, with higher drug loading and no escape of drug from the core (Salvi and Pawar, 2019). NLCs can be classified into type I (disordered, imperfect matrix), type II (oily nanocompartments) and type III (solid non-crystalline amorphous matrix) (Selvamuthukumar and Velmurugan, 2012). Researchers have compared SLNs and NLCs and determined better efficacy of NLCs in terms of drug release, stability profile, percent entrapment efficiency, and tissue penetration (Das et al., 2012; Balguri et al., 2016).

There are many different formulation methods for NLCs, which include high-pressure homogenization (HPH), solvent emulsification evaporation method, solvent emulsification diffusion method, solvent injection method, microemulsion method, double emulsion technique, ultrasonication or high-speed homogenization, phase inversion method, membrane contractor technique, supercritical fluid (SCF) method, and hot-melt extrusion (HME) technology (Elmowafy and Al-Sanea, 2021; Garg et al., 2022). Recently, numerous NLCs formulations have been used for therapeutic applications, particularly for the delivery of poorly soluble drugs, due to their biocompatible nature and use of lipids (Teixeira et al., 2017). Several researchers have developed NLC formulations against diverse types of cancer including lung, brain, and breast cancer. Wang et al. prepared paclitaxel and DOX-loaded NLCs and observed a synergistic effect of the two chemotherapy drugs in a lung cancer model, with less systemic cytotoxicity, and also enhanced *in vitro* efficacy against NCL-H460 lung cancer cells. (Wang et al., 2016). In another study, Ong et al. prepared NLCs loaded with thymoquinone and administered them orally to 4T1 metastatic mammary carcinoma tumor-bearing mice. The formulation showed improved anticancer efficacy and increased survival rate compared to oral thymoquinone alone. (Ong et al., 2018). Sabzichi et al. prepared NLCs loaded with vitamin D and DOX, (Sabzichi et al., 2017), and found that the co-administration of vitamin D with DOX using the NLC carrier improves therapeutic efficacy in MCF-7 breast cancer cells. Curcumin-NLC formulations have demonstrated improved anticancer efficacy in A172 glioblastoma cells and improved efficacy with lower doses for an *in vivo* in A172 xenograft mice model (Chen et al., 2016). Some groups have incorporated phytochemicals for cancer treatment into NLCs, for instance, Gadag et al. developed NLCs containing resveratrol and delivered them into breast tissue using microneedle arrays, resulting in enhanced *in vivo* permeation as well as improved *in vitro* antitumor activity against MDA-MB-231 breast cancer cells. (Gadag et al., 2021). Recently, Sadeghzadeh et al. prepared NLCs loaded with umbelliprenin and coated with folic acid-bound chitosan to target breast cancer cells. Experiments in tumor-bearing mice confirmed good anti-angiogenesis and anti-tumor effects, with over 75% volume reduction in less than a month. (Sadeghzadeh et al., 2023). Some more detailed examples of NLC formulations are shown in Table 2.

**TABLE 2:** Examples of nanostructured lipidic carrier formulations.

Nanostructured lipid composition (NLC)	Targeting (agents)	Drug	Size (nm)	Remarks	References
steric acid, oleic acid, capric/caprylic triglycerides (MCT), monoglyceride (MGE)		4-dedimethylamino sancycline (CMT-3)	130–200	CMT-3/NLC showed cytotoxicity against HeLa cells, facilitated cell uptake and reached to cytoplasm	Yang et al. (2013)
Compritol^®^ 888 ATO (solid lipid),Capryol™ 90 (liquid lipid), Palmitic acid (solid lipid), stearic acid (solid lipid), oleic acid (liquid lipid), and ethyl oleate (liquid lipid), Soybean oil (liquid lipid)		triptolide (TP)	225–235	TP-NLCs decreased fluctuations of TP in plasma and were a better carrier of TP compared to TP-SLNs for oral delivery application	Zhang et al. (2014)
cholesterol, Compritol^®^, poloxamer 188,Stearylamine, soy lecithin, oleic acid, tween 80	Transferrin (TF)	Artemisinin (ART)	130–150	TF-ART-NLCs has the potential to deliver antimalarial drug ART in brain tumors and malaria	Emami et al. (2018)
Glycerin monostearate (GM), Oleic acid (OA),Phosphatidylcholine (PC)	folic acid, PEG	Docetaxel	30–40	DTX. FA-NLC showed remarkable tumor inhibition efficacy in HeLa tumor-bearing BALB/c mice	Li et al. (2020)
Softisan 154 (S154), olive oil, lecithin, l-α-phosphatidylcholine, polysorbate 80,sorbitol, thimerosal		Tamoxifen (TAM)	40–50	TAM-NLC showed cytotoxic effect for MCF-7 and 4T1 cell lines comparable to free drug. These can be used as therapeutic agents	How et al. (2013)
Tristearin, stearic triglyceride (tristearin), Miglyol 812, poloxamer 188, caprylic/capric triglycerides (Miglyol)		bromocriptine	190–270	BC–NLC were able to markedly diminish motor deficit in 6-OHDA hemi-lesioned rats	Esposito et al. (2012)
Glycerol monostearate (GM), soybean phosphatidylcholine (SPC), oleic acid	TopII	Etoposide (VP16) (ETP)	70–100	ETP-NLCs exhibited *in vivo* antitumor effect in SGC7901 cells xenografts and gastric cancer animal model	Jiang et al. (2016)
glyceryl monostearate, soya lecithin, soybean oil,Tween-80	Thymidylate synthase/Hyaluronic acid	Cisplatin + 5-fluorouracil FU	90–180	HA-FU/C-NLC exhibited strongest antitumor activity in mice bearing BGC823 human GC xenografts	Qu et al. (2015)
stearic acid (SA), oleic acid (OA), and lecithin, Poly (ethylene glycol)–distearoylphosphatidylethanolamine (PEG–DSPE)	EGFRvIII monoclonal antibody (MAb)	Doxorubicin	130–250	Anti-EGFRvIII MAb-targeted NLC increased the cytotoxic effect of doxorubicin on HC2 20d2/c cells	Abdolahpour et al. (2018)
Capryol^®^ 90, Labrasol^®^, and Compritol^®^888, Miglyol^®^ 840, Poloxamer^®^ 407, Oleic acid		Temazepam	240–350	Temazepam-loaded nanostructured lipid carriers (NLCs) facilitated drug transport to the brain	(E. Eleraky et al., 2020)

### 2.5 Lipid polymer nanohybrids

The two most studied and utilized nanodelivery systems are liposomal and polymeric nanoformulations, with each platform having its own intrinsic limitations and advantages. Lipid-polymer nanoparticle hybrids (LPNHs) have been developed to exploit and combine the positive characteristics of these nanosystems, while trying to minimize their unique disadvantages. LPNHs consist of 1) a lipophilic polymeric core that can incorporate hydrophobic drugs, 2) a lipid shell surrounding the polymeric core and serving as a barrier for drug retention within the core, and 3) a hydrophilic polymer stealth layer at the circumference of the lipid shell, typically PEG-containing, which improves stability and prolongs circulation time (Whitlow et al., 2016). Also, the polymers used in LPNHs can be synthetic or natural with different degrees of crosslinking, and this choice can be customized to enable control of the release profile of the incorporated drug (Petralito et al., 2014).

The preparation of LPNHs can follow a one-step approach or a two-step approach. In the one-step procedure, LPHNs are prepared as part of a single process using nanoprecipitation and self-assembly to add a single lipid layer shell onto the polymeric core. A water-miscible solvent such as acetonitrile is used to dissolve polymers and lipophilic drugs, whereas lipids and PEG-lipid derivatives are dissolved in aqueous solution. The solubility of phospholipids is enhanced by adding a small amount of water-miscible organic solvents to the aqueous solution. Next, dropwise addition of polymer solution to the lipid aqueous phase by diffusing the organic solution into the aqueous solution takes place, which causes the polymer to precipitate into nanoparticles. The lipids accumulate on the surface of polymer nanoparticles through hydrophobic interactions, giving rise to LPNHs. Two-step methods, on the other hand, involve separate preparation of the polymer core and the lipid shell. First, the polymer core is prepared using emulsion (either single or double) and high-pressure homogenization (breaking polymer mixtures or melted polymers into little globules once they cross a very thin nozzle), and then liposomes are prepared separately using sonication or an extrusion technique. Once these two formulations are prepared, they are mixed at specific molar ratios by needle extrusion or a simple vortexing method, finally resulting in LPHNs.

LPHNs exhibit excellent drug loading and stability for *in vivo* use (Zhang et al., 2008). Their unique composition with a stealth polymer coating affords the advantage of improved pharmacokinetics, increased effective dose at target site, and enhanced steric stabilization. The selection of specific polymer compositions can help control release, and these nanohybrid systems can be used to target delivery to localized tissues in cancer treatment (Zhang and Zhang, 2010). Chan et al. designed a paclitaxel-based “nanoburr” system made of a paclitaxel-coupled PLA core and a shell composed of lecithin/DSPE-PEG, coupled with a targeting peptide. The researchers found that these LPHNs specifically accumulated in damaged vasculature in rats, and demonstrated the ability to release drug over the span of 2 weeks (Chan et al., 2010). In general, the multilayered structure of LPHNs also lends itself to the combination of different agents to create multifunctional carriers, delivery of synergistic agents, or application of theranostic approaches. Wang et al. designed a LPHN system that can be used for chemotherapy and radiotherapy, by encapsulating docetaxel in the core and adding a radioisotope to the shell (Wang et al., 2010). Yang et al. prepared magneto-polymeric nanohybrids (HER-MMPNs) with the chemotherapy agent trastuzumab (Herceptin) and monodispersed magnetic nanocrystals (Fe_3_O_4_) co-encapsulated into an amphiphilic block copolymer. This formulation slowed tumor growth rate in an *in vivo* breast cancer NIH3T6.7 cell xenograft (Yang et al., 2007), and the magnetic nanocrystals enabled MRI imaging of the tumor, thus creating a theranostic system. Shukla et al. developed spermine (SPM)-tethered lipo-polymeric hybrid nanoconstructs with cell surface heparan sulfate proteoglycan (HSPG) and loaded with DOX and genistein (an anti-angiogenic agent). These LPHNs provided specific intracellular localization and pH-dependent release in the acidic tumor environment, so that DOX and genistein were released at the appropriate site for synergistic action (Shukla et al., 2020). More recently, Riadi et al. developed baicalin-loaded lipid–polymer hybrid nanoparticles to specifically target colorectal cancer. Biodistribution studies showed preferential uptake to the colon compared to a baicalin suspension, and enhanced ability to reduce elevated levels of liver enzymes, (Riadi et al., 2023), however, tumor-bearing studies would need to be completed for a comprehensive understanding of biodistribution and effects.

Some more detailed examples of lipid polymer nanohybrid formulations are shown in Table 3.

**TABLE 3:** Examples of lipid polymer nanohybrid formulations.

Lipid-polymer composition	Targeting (agents)	Drug	Size (nm)	Remarks	References
PLGA-PEG-PLGA triblock copolymer, with PLGA = Poly (D, L-lactide-co-glycolide) cholesterol, lecithin		Salidroside	150–160	Drug loaded-core shelled LPNPs showed improved *in vitro* antitumor activity of Salidroside in PANC-1 and 4T1 cancer cell lines	Fang et al. (2014)
PLGA, Soybean lecithin, Poly (ethylene glycol)–distearoylphosphatidylethanolamine (PEG–DSPE)		2′-Deoxy-5-azacytidine (DAC) + Doxorubicin	50–120	Co-loaded lipid–polymer nanoparticles showed enhanced toxicity in *in vitro* MB231 cells	Su et al. (2013)
poly (β-amino ester) (PBAE),Poly (lactide-co-glycolide) (PLGA),1,2-dioleoyl-sn-glycero-3-phosphocholine (DOPC), 1,2-dioleoyl-3-trimethylammonium-propane (chloride salt) (DOTAP), 1,2-distearoyl-sn-glycero-3-phosphoethanolamine-N-[methoxy-(polyethyleneglycol)-2000] (ammonium salt) (DSPE-PEG), 1,2-distearoyl-sn-glycero-3-phosphoethanolamine-N-[methoxy-(polyethyleneglycol)-2000-N′-carboxyfluorescein] (ammonium salt) (DSPE-PEG-CF), and 1,2-dioleoyl-sn-glycero-3-phosphoethanolamine-N-(lissamine rhodamine B sulfonyl) (ammonium salt) (DOPE-rhodamine)		mRNA	350–510	Lipidic polymer NP delivery system showed enhanced cytosolic delivery of mRNA in C5BL/6J mice with reduced toxicity	Su et al. (2011)
phosphatidylcholine (PC), and 1,2-distearoyl-*sn*-glycero-3-phosphoethanolamine-N-[methoxy (polyethyleneglycol)-2000] (DSPE-PEG)	Hyaluronic acid (HA)	Ginsenoside Rg3	100–180	Pharmacokinetic study in rats showed reduced *in vivo* clearance of (S)-Rg3 with nanocomplex based delivery system	Lee et al. (2014)
Poly (maleic anhydride-alt-1-octadecene), Vitamin E acetate (VEA)		Rhodamine, BODIPY	two population 400–600 and 80–100	20 wt% of the polymer was an optimal ratio for obtaining stable HNPs by nanoprecipitation	Bou et al. (2020)
PLGA, lecithin, and 1,2-distearoyl-Sn-glycero-3-phosphoethanolamine-N-methoxy (polyethylene glycol)-2000 (DSPE-PEG 2000)		DOX hydrochloride	173–208	Doxorubicin-loaded lipidic nanocarriers showed greater antiproliferation effects in MDA-MB231 and PC3 cells as compared to free doxorubicin	Tahir et al. (2019)
PLGA, 1,2-didodecanoyl-*sn*-glycero-3-phosphocholine synonyms: 1,2-dilauroyl-*sn*-glycero-3-phosphocholine (DLPC),1,2-distearoyl-sn-glycero-3-phosphoethanolamine-N-[methoxy (polyethylene glycol)-2000] (DSPE-PEG2k)	Folate	Docetaxel	200–300	LPNPs exhibited controlled and targeted delivery of anticancer drugs in *in vitro* studies for MCF7 cells	Liu et al. (2010)
PLGA, lecithin, DSPE−PEG (1,2-distearoyl-sn-glycero-3-phosphoethanolamine-N-carboxy (polyethylene glycol)2000)	A10 aptamer	Docetaxel	66–90	Self- assembled aptamer-based LPNPs specifically bind to prostate specific membrane antigen (PSMAPC3) in prostate adenocarcinoma	Zhang et al. (2008)
Chitosan, Lipid (Lipoid S75)		Cisplatin	181–245	LPNPs pharmacokinetic study in rabbits showed controlled delivery of cisplatin with improved mean residence time and half-life	Khan et al. (2019)
PLGA, 1,2-distearoyl-sn-glycero-3-phosphoethanolamine-N-carboxy (polyethylene glycol) maleimide (DSPE-PEG-maleimide), lecithin	Anti-EGFR antibody	Doxorubicin	100–120	Anti-EGF receptor antibody tagged LPNPs showed significantly enhanced antitumor activity against HCC *in vivo*	Gao et al. (2014)

### 2.6 Supramolecular nanolipidic structures

Traditional lipidic carriers are used as delivery vehicles to carry therapeutic agents to tumors, and to reduce off-target uptake and improve *in vivo* pharmacokinetics. In this case, lipids have a structural role. They are simply the building blocks and the carrying vessel, but they do not serve an inherent therapeutic or theranostic purpose beyond their cargo (Huynh and Zheng, 2014). Coupling lipids with other organic molecules has led to the creation of lipid-based supramolecular structures, also known as supramolecular nanolipidic structures (SMNLS), which have additional functionality such as assisting drug release, or serving theranostic or synergistic purposes.

An example is the combination of heterocyclic organic molecules such as porphyrins with lipid formulations. By themselves, porphyrins can be used for imaging and photodynamic therapy in cancer. For instance, the molecule benzoporphyrin derivative monoacid (BPD-MA) is a strong photosensitizer. The photophysical properties of porphyrins, including higher absorption wavelength and better quantum yield, coupled with low *in vivo* toxicity, make them good candidates for clinical photosensitizing applications in cancer. However, poor lipophilicity is a major bioapplication barrier for most of the porphyrins. Liposome carriers can be used to try to overcome this issue, and a BPD-MA liposomal formulation has shown enhanced *in vivo* photosensitizer efficiency and has already been marketed as Visudyne^®^. However, low loading capacity of porphyrins into liposomes limits their application to imaging only.

To overcome this limitation, the creation of SMNLS has attempted to integrate the carrying capacities of lipids along with new abilities imparted by conjugation to other molecules. Komatsu et al. conjugated phospholipids with porphyrin in a 4:1 phospholipid: porphyrin molecule ratio, and prepared a liposome-like formulation with enhanced properties such as strong fluorescence, red-shifted band, and small lifespan of triplet state (Komatsu et al., 2002). Lovell et al. prepared a different porphyrin-lipid formulation through conjugation of porphyrin with glycerol in a 1:1 ratio. In these structures, porphyrins are still able to fluoresce but are also densely packed, which gives raise to enhanced heat generation properties that are limited in other formulations. (Lovell et al., 2011). Metal doping can also make these structures amenable to MRI detection. (Huynh and Zheng, 2014). Thus, the combination of porphyrins and lipids into supramolecular structures provides heightened functionality for multipurpose uses, while still maintaining the advantage of having a lipidic carrier structure for improved delivery and stability.

In a drug release application, Wang et al. functionalized phospholipids with complementary nucleic base pairs, so that the heads were functionalized with uridine and the tails with adenosine. These systems easily self-assemble into liposome-like formulations that are responsive to pH triggers. The researchers loaded these structures with DOX and observed that they were successfully internalized into tumor cells and were much more efficient in releasing DOX when triggered by an acidic environment, compared to conventional DOX-loaded liposomes (Wang et al., 2015). The DOX-loaded SMNLs also demonstrated higher *in vitro* cytotoxicity in MCF-7 breast cancer cells compared to conventional DOX-loaded liposomes. In an *in-vivo* breast cancer model, the DOX-loaded SMNLs showed prolonged plasma half-life and increased tumor site accumulation compared to free DOX or conventional DOX-loaded liposomes, as well as higher reduction in tumor volume.

## 3 Looking forward: From bench to clinic

A clear advantage of nanoformulations from a clinical perspective in the treatment of cancer is the ability to deploy not only chemotherapeutic agents, but also immunotherapy, genetic-based approaches, or even phytomedicine formulations. (Handa et al., 2021; Gupta et al., 2022). The shared vision of researchers in the field is that the development of customizable nanoformulations will open a new era of personalized medicine; a reality where cancer management can be tailored to the patient’s type of cancer, stage, biomarker and/or genetic profile through the rational design of nanosystems that specifically and effectively address desired targets. There is also the potential to make use of the multifunctionality of these formulations to streamline management, for example, by decreasing the number of interventions a patient must receive by using combinational therapies or deploying theranostic approaches that allow for real-time monitoring, treatment, and follow-up. This could result in enhanced patient clinical outcomes as well as enhanced perceived quality of life for patients undergoing cancer treatment, and potentially reduced therapeutic costs as efficacy could be obtained at lower doses.

Among nanopharmaceuticals developed to treat cancer, 56% are lipid-based nanoformulations (Rodríguez et al., 2022), likely because of their ability to control release, high loading efficiency, potential for multifunctional use, impact on drug-resistant cancer lines, biocompatibility, and safety profile which makes them more attractive to regulators. Review of the CAS content collection reveals over 45,000 patents related to lipid-based nanoformulations in the last 20 years, (Tenchov et al., 2021), and our updated Google Patent search for worldwide patents related to lipid-based nanoformulations in cancer between 2019 and 2023 returned over 28,000 results including varied lipid-based nanoformulations, drug delivery and multifunctional platforms, and pharmaceutical as well as nutraceutical approaches. An interesting trend that was observed in the past year (2022–2023) is the increasing number of patents where a lipid-based nanoformulation is combined with DNA or RNA material, often related to immunotherapy approaches.

**FIGURE 2:**
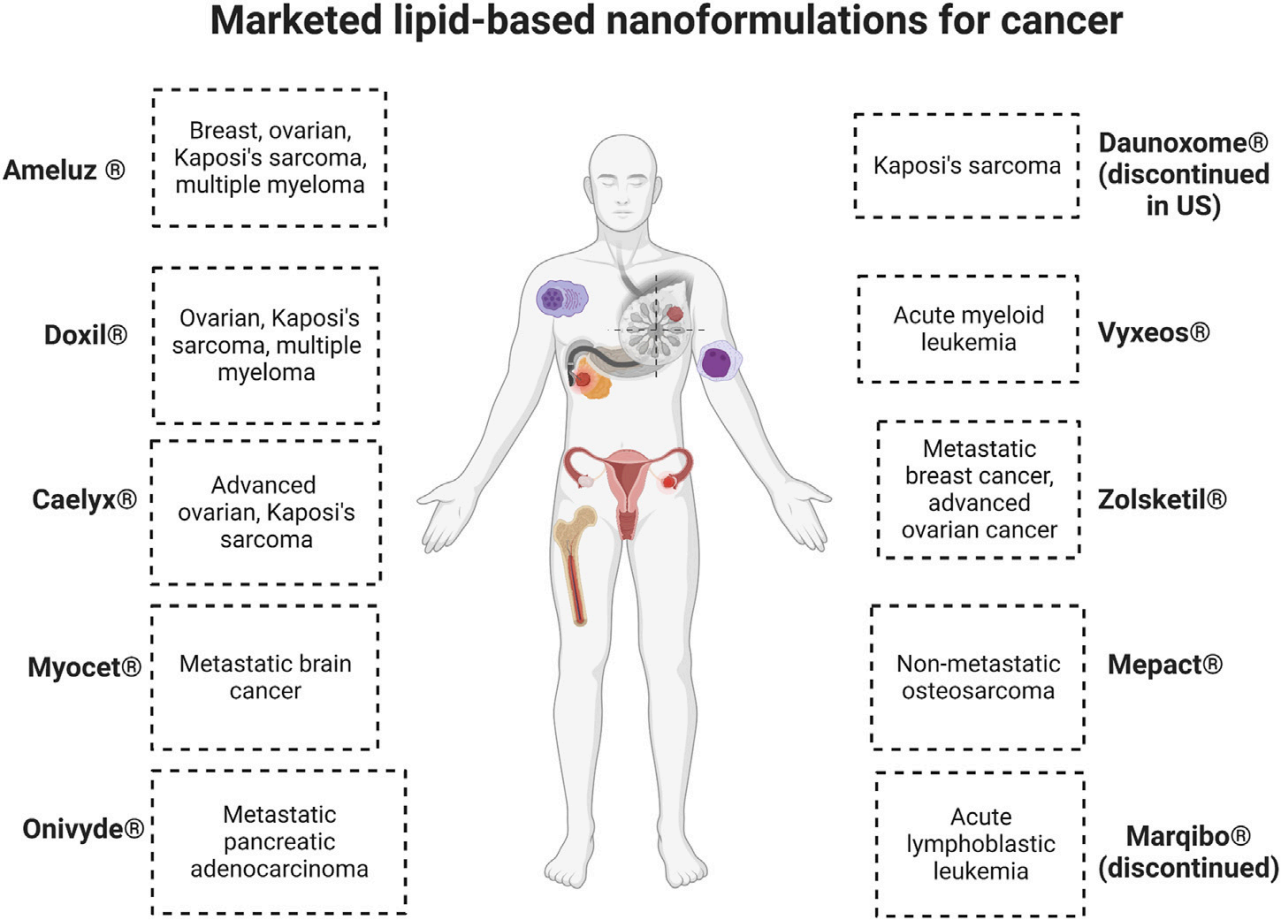
Commercialized lipid-based nanoformulations. Created with BioRender.com.

Lipid nanoformulations have evolved into the clinical field, with many advancing to clinical trials and some reaching the market. In clinical anti-cancer use, some of these nanodrugs have demonstrated prolonged circulation time and increased plasma-half life, enhanced uptake in tumors, and improved efficacy with reduced off-site toxicity compared to conventional drug formulations. (Loo et al., 2022; Zeng et al., 2023). There are many lipid-based nanoformulations that are currently in clinical trials spanning end times between 2023 and 2032, and Table 4 shows some examples of currently ongoing clinical trials. Lipid-based nanoformulations for cancer treatment that have obtained market approval in the United States and/or Europe include nanoformulations with single or combinational loads of daunorubicin (Daunoxome^®^, Vyxeos^®^), doxorubicin (Doxil^®^, Myocet^®^, Caelyx^®^, Zolsketil^®^), cytarabine (Vyxeos^®^), irinotecan (Onivyde^®^), mifamurtide (Mepact^®^), 5-aminolevulinic acid (Ameluz ^®^), and vincristine (Marqibo^®^) with applications in different types of sarcomas, lymphoma, leukemia, pancreatic cancer, colorectal cancer, ovarian cancer, breast cancer, myeloma, and skin cancer. (Abdellatif and Alsowinea, 2021; Tenchov et al., 2021; Rodríguez et al., 2022; Taléns-Visconti et al., 2022). Figure 2 illustrates their specific applications in cancer.

**TABLE 4:** Some examples of clinical trials involving lipid-based nanoformulations for cancer treatment.

Particle type/drug		Conditions/ Diseases	Identifier / Status	Details	
Lipid nanoparticles Drug: WGI-0301		Advanced Solid Tumors	NCT05267899(Ph1):Recruiting	https://clinicaltrials.gov/ct2/show/NCT03823989	
				Actual Study Start Date:	1-Aug-22
				Estimated Primary Completion Date:	12-Sep-23
				Estimated Study Completion Date:	26-Dec-23
Lipid nanoparticles Biological: quaratusugene ozeplasmid Drug: pembrolizumab Drug: docetaxel Drug: ramucirumab		Non-Small Cell Lung Cancer	NCT05062980 (Ph 1/2): Recruiting	https://clinicaltrials.gov/ct2/show/NCT05062980	
				Actual Study Start Date:	30-Mar-22
				Estimated Primary Completion Date:	May-25
				Estimated Study Completion Date:	May-26
Lipid nanoparticles Biological: mRNA-2752, Biological: Durvalumab		Dose Escalation: Relapsed/Refractory Solid Tumor Malignancies or Lymphoma Dose Expansion: Triple Negative Breast Cancer, HNSCC, Non-Hodgkin’s, Urothelial Cancer, Immune Checkpoint Refractory Melanoma, and NSCLC Lymphoma	NCT03739931(Ph1):Recruiting	https://clinicaltrials.gov/ct2/show/NCT03739931	
				Actual Study Start Date:	27-Nov-18
				Estimated Primary Completion Date:	30-Jan-23
				Estimated Study Completion Date:	30-Jan-23
Lipid nanoparticles Biological: quaratusugene ozeplasmid Drug: osimertinib		Carcinoma, Non-Small Cell Lung	NCT04486833 (Ph1)((Ph2): Recruiting	https://clinicaltrials.gov/ct2/show/NCT04486833	
				Actual Study Start Date:	3-Sep-21
				Estimated Primary Completion Date:	Dec-24
				Estimated Study Completion Date:	Dec-25
Lipid nanoparticles	Drug: OTX-2002	Hepatocellular Carcinoma	NCT05497453(Ph1): Recruiting	https://clinicaltrials.gov/ct2/show/NCT05497453	
	Drug: Tyrosine kinase inhibitor One	Solid Tumor		Estimated Study Start Date:	19-Aug-22
	Drug: Tyrosine kinase inhibitor Two	Hepatocellular Carcinoma Non-resectable		Estimated Primary Completion Date:	Jun-25
	Drug: Checkpoint Inhibitor, Immune	Hepatocellular Carcinoma Recurrent Hepatocellular Cancer Liver Cancer		Estimated Study Completion Date:	Dec-28
Liposomes Pegylated Liposomal Doxorubicin + SL-172154 Mirvetuximab + SL-172154		Liver Cancer, Non-Resectable; Platinum-resistant Ovarian Cancer; Fallopian Tube Cancer; Epithelial Ovarian Cancer; Ovarian Cancer; Platinum-Resistant Fallopian Tube Carcinoma; Platinum-Resistant Primary Peritoneal Carcinoma; Primary Peritoneal Carcinoma	NCT05483933 (Ph1): Recruiting	https://clinicaltrials.gov/ct2/show/NCT05483933	
				Actual Study Start Date:	18-Aug-22
				Estimated Primary Completion Date:	Jul-24
				Estimated Study Completion Date:	Apr-25
Liposomes BP1001-A (Liposomal Grb2 Antisense Oligonucleotide)BP1001-A (Liposomal Grb2 Antisense Oligonucleotide) with paclitaxel		Solid Tumor, Adult Carcinoma, Ovarian Epithelial Fallopian Tube Neoplasms Endometrial Cancer Peritoneal Cancer Solid tumor	NCT04196257 (Ph1): Recruiting	https://clinicaltrials.gov/ct2/show/NCT04196257	
				Actual Study Start Date:	19-Aug-22
				Estimated Primary Completion Date:	Jul-24
				Estimated Study Completion Date:	Oct-24
Liposomes Liposomal HPV-16 E6/E7 Multipeptide Vaccine PDS0101 Pembrolizumab		Clinical Stage III HPV-Mediated (p16-Positive) Oropharyngeal Carcinoma AJCC v8 Human Papillomavirus-Related Carcinoma Locally Advanced Oropharyngeal Carcinoma Pathologic Stage III HPV-Mediated (p16-Positive) Oropharyngeal Carcinoma AJCC v8 Stage IVA Oropharyngeal (p16-Negative) Carcinoma AJCC v8	NCT05232851 (Ph1,Ph2): Recruiting	https://clinicaltrials.gov/ct2/show/NCT05232851	
				Actual Study Start Date:	7-Mar-22
				Estimated Primary Completion Date:	1-Jul-23
				Estimated Study Completion Date:	1-Jul-24
Liposomes Drug: LTLD Procedure: MR-HIFU induced hyperthermia Drug: Cyclophosphamide		Metastatic Breast Cancer; Breast Cancer/Neoplasms; Stage IV Breast Cancer; Invasive Ductal Carcinoma of Female Breast; Adenocarcinoma Breast	NCT03749850 (Ph 1): Recruiting	https://clinicaltrials.gov/ct2/show/NCT03749850	
				Estimated Study Start Date:	Mar-21
				Estimated Primary Completion Date:	Nov-22
				Estimated Study Completion Date:	Nov-22
Liposomes Drug: CPX-351 Drug: Midostaurin Drug: Busulfan Drug: Melphalan Drug: Fludarabine Biological: CD34+ selected allogeneic stem cell transplant from an HLA-compatible donor		Acute Myeloid Leukemia	NCT04982354 (Ph 1) (Ph2):Recruiting	https://clinicaltrials.gov/ct2/show/NCT04982354	
				Actual Study Start Date :	5-Jul-22
				Estimated Primary Completion Date :	1-Aug-31
				Estimated Study Completion Date :	1-Aug-32
Liposomes Drug: BP1002; Liposomal Bcl-2 Antisense Oligodeoxynucleotide Drug: Decitabine (in combination with BP1002)		Acute Myeloid Leukemia, in Relapse Acute Myeloid Leukemia Refractory	NCT05190471 (Ph1): Recruiting	https://clinicaltrials.gov/ct2/show/NCT05190471	
				Actual Study Start Date:	16-Aug-22
				Estimated Primary Completion Date:	Mar-24
				Estimated Study Completion Date:	Sep-24

Out of these drugs, two of them which received initial or accelerated approval were retired post-marketing for varied reasons. Marqibo^®^ was discontinued in the Unites States because the post-marketing clinical trial required to ascertain clinical benefit was not completed, and there were issues achieving sufficient levels of patient recruitment. Discontinuation was requested in 2021 and officially approved in May 2022. (FDA, 2022a). Daunoxome^®^ was discontinued in the Unites States as of December 2021, along with 17 other medications marketed by the same company. (FDA, 2021). Both of these medications were designated as orphan drugs, which are pharmaceuticals to treat rare medical conditions so that profitability within 7 years of approval was not feasible without assistance from the government.

**FIGURE 3:**
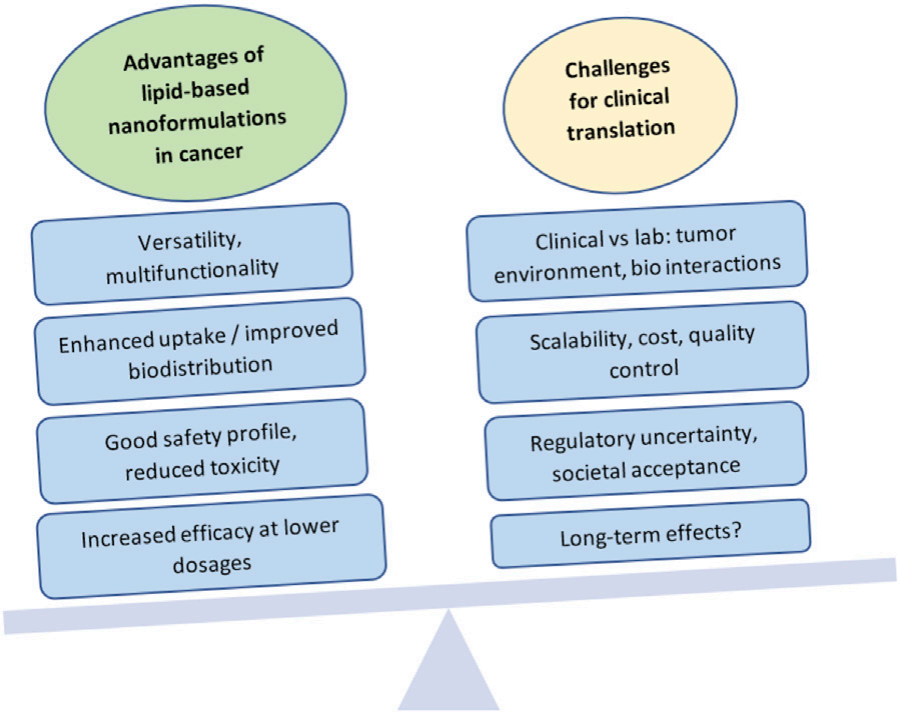
Advantages and challenges in the clinical translation of lipid-based nanoformulations for cancer management. Created with BioRender.com.

This highlights the point that, although the field is showing much promise, there have been many challenges to commercialization and clinical success for these formulations (Figure 3). Some of these hurdles include attaining large-scale production while controlling design specifications, lack of full understanding of long-term toxicity, translational variation from *in vivo* animal models to humans (such as changes in immune system interaction, biodistribution, or bioavailability), pricing and equitable access, profitability, and societal acceptance. (Metselaar and Lammers, 2020).

What works in the lab does not always work in clinical trials, and the challenges in translation are evident. Research paradigms emphasize a formulation-driven method, where new formulations are created first, and then their efficacy and safety are tested. (Thapa and Kim, 2023). Unfortunately, while many *in vitro* experiments are successful, formulations may then turn out to be inefficacious in animal models or in clinical trials. It is estimated that about 20%–25% of preclinical studies are translated into clinical applications, and the success rate of clinical trials thereafter drops from 94% in phase I to a disappointing 14% in phase III, due to failure to demonstrate clinical efficacy and good safety profiles. (Fogel, 2018; Đorđević et al., 2022).

The inherent heterogeneity of cancer can be a significant therapeutic challenge for nanoformulations. (Shan et al., 2022). The complex pathophysiology of tumors, as well as the multifaceted biological interactions between drugs and body systems, require an intricate understanding of the *in vivo* mechanisms that affect drug uptake, interactions with biological moieties and the immune system, cell responses, and tumor adaptation processes such as the development of multi-drug resistance, among others. Therefore, in developing formulations with good potential for translation, it is critical to carefully consider the end goal and create designs with this goal in mind: full considerations of biocompatibility, toxicity, therapeutic index, route of administration, interaction with plasma or immune components, and pharmacokinetics. (Đorđević et al., 2022). These aspects should not come after the design, but rather should constitute an integral part of design considerations. Although much of this information will not be truly available until researchers and clinicians carry out *in vivo* studies, preliminary reflection should be part of intentional design and should be carried through the pre-clinical phase in order to maximize the possibilities of successful translation.

Even when a drug shows promise, one significant challenge has been scaling up manufacturing processes to translate what is done in small batches in the lab, into a product that can be mass-marketed, reach the bedside, and stay in market. When considering lipid-based formulations, factors such as particle size, polydispersity, structure, loading or encapsulation efficiency, and stability become more difficult to control and standardize at large scales. If fabrication is not reproducible, small changes can completely alter the properties, biodistribution, effectiveness and safety of the nanoformulation. (Taléns-Visconti et al., 2022). Therefore, unique manufacturing practices and quality control processes must be developed to successfully mass produce these formulations, and ensuring that this can be done at a reasonable cost is crucial (Thapa and Kim, 2023).

Clinical translation and public acceptance is also hindered by the lack of consensus on regulatory aspects of nanoformulation development, testing, commercialization, and post-market monitoring (Đorđević et al., 2022). Although efforts have been made to develop international regulations, the regulatory landscape remains generally fractured, with each country or region adopting its own standards, if any. For instance, in the United States, the Food and Drug Administration (FDA) published a guidance document in 2017 intended “For Drug Products, Including Biological Products, that Contain Nanomaterials” and has since been updating the guidance based on industry and stakeholder comments, with the latest version approved in April 2022 (FDA, 2022b). Although it is non-binding, and the FDA takes a secondary role of a consultant or post-marketing safety monitor, the document covers important aspects that must be considered from the point of view of the unique characteristics of nanoformulations, including among others: potential risk evaluation, structural and physicochemical characterization, quality testing, manufacturing process controls, excipient safety and stability, Absorption-Distribution-Metabolism- Excretion (ADME) and route of administration, clinical development, immunogenicity, and environmental impact. In the document, the FDA attempts to emphasize the differential aspects of nanoformulations compared with conventional drug approaches, for example, considering how toxicology may be impacted or changed when an existing drug product is modified to make a nanoformulation. However, to this day, nanomedicines often go through an expedited approval process if they are derived from an existing compound, and the non-binding nature of the guidelines makes them less valuable (Đorđević et al., 2022). In order to protect patient safety and address societal concerns about the unknown aspects of nanoformulations, a crucial step is the development of a clear regulatory framework, ideally as part of an international collaborative effort. Undoubtedly, there is still much research left to do in order to reach the point when nanoformulations become the benchmark for patient management in cancer, but the possibilities of lipid-based nanoformulations are very promising.
